# Modeling Study of Si_3_N_4_ Waveguides on a Sapphire Platform for Photonic Integration Applications

**DOI:** 10.3390/ma17164148

**Published:** 2024-08-22

**Authors:** Diandian Zhang, Shui-Qing Yu, Gregory J. Salamo, Richard A. Soref, Wei Du

**Affiliations:** 1 Department of Electrical Engineering and Computer Science, University of Arkansas, Fayetteville, AR 72701, USA; dz010@uark.edu (D.Z.); syu@uark.edu (S.-Q.Y.); salamo@uark.edu (G.J.S.); 2 Institute for Nanoscience and Engineering, University of Arkansas, Fayetteville, AR 72701, USA; 3 Department of Engineering, University of Massachusetts at Boston, Boston, MA 02125, USA; soref@rcn.com

**Keywords:** waveguides, photonic integrated circuits, sapphire

## Abstract

Sapphire has various applications in photonics due to its broadband transparency, high-contrast index, and chemical and physical stability. Photonics integration on the sapphire platform has been proposed, along with potentially high-performance lasers made of group III–V materials. In parallel with developing active devices for photonics integration applications, in this work, silicon nitride optical waveguides on a sapphire substrate were analyzed using the commercial software Comsol Multiphysics in a spectral window of 800~2400 nm, covering the operating wavelengths of III–V lasers, which could be monolithically or hybridly integrated on the same substrate. A high confinement factor of ~90% near the single-mode limit was obtained, and a low bending loss of ~0.01 dB was effectively achieved with the bending radius reaching 90 μm, 70 μm, and 40 μm for wavelengths of 2000 nm, 1550 nm, and 850 nm, respectively. Furthermore, the use of a pedestal structure or a SiO_2_ bottom cladding layer has shown potential to further reduce bending losses. The introduction of a SiO_2_ bottom cladding layer effectively eliminates the influence of the substrate’s larger refractive index, resulting in further improvement in waveguide performance. The platform enables tightly built waveguides and small bending radii with high field confinement and low propagation losses, showcasing silicon nitride waveguides on sapphire as promising passive components for the development of high-performance and cost-effective PICs.

## 1. Introduction

Due to their superior performance, energy efficiency, and versatility across a wide range of applications, photonic integrated circuits (PICs) are becoming increasingly vital in modern technology. Currently, PICs are mainly implemented using InP, Silicon Photonics, and TriPleX™ technologies. The InP platform is widely used in devices such as light sources, modulators, and photodetectors [1,2,3]. For this reason, the InP platform has inherent potential in PICs [2,3,4,5,6]. Nevertheless, the performance of optical waveguides based on III–V materials, an indispensable component in PICs, remains unsatisfactory, with propagation losses reaching orders of magnitude higher than those of silicon or silicon nitride waveguides [7,8,9,10,11,12]. Another limiting factor is CMOS incompatibility, which further constrains its scale-up manufacturing. TriPleX™ technology utilizing silicon nitride waveguides demonstrates exceptional performance due to its ultra-low loss, CMOS-compatible fabrication process, and low-cost volume production [13,14,15,16,17,18]. However, this technology does not directly support active devices, such as lasers, modulators, and photodetectors. Consequently, a hybrid approach incorporating another platform is necessary to complement the limitations of this technology [19,20,21,22]. Silicon photonics, leveraging the mature silicon IC manufacturing and high-quality SOI planar waveguide circuits, has garnered significant interest over the last two decades [23,24]. The indirect bandgap nature of Si and Ge requires hybrid integration with III–V materials for efficient light sources. As a result, issues of lattice mismatch and thermal expansion mismatch must be addressed to obtain high-quality materials.

**Figure 1:**
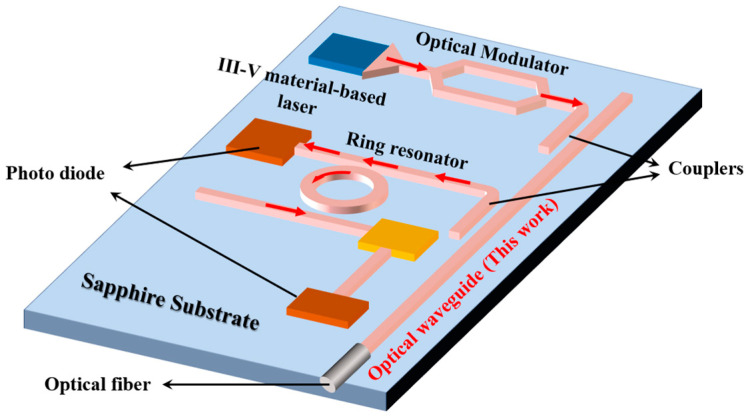
Schematic of the sapphire PIC.

Driven by the current situation, the PIC-on-sapphire platform has been proposed recently, as shown in Figure 1. Thanks to rapid advancements in material growth technology, III–V materials can now achieve epitaxial growth on sapphire substrates [25,26,27,28,29]. It is worth noting that sapphire exhibits a coefficient of thermal expansion (CTE) closely matching that of III–V materials, as shown in Table 1; therefore, the sapphire platform could inherit nearly all the advantages of the III–V platform [25,30,31,32]. This characteristic gives the sapphire platform inherent advantages for devices such as light sources, modulators, photodetectors, and more. The sapphire platform also supports passive devices and silicon-on-sapphire (SOS) circuits, enabling a fully integrated solution that includes a comprehensive set of components, such as light sources, modulators, light detectors, passive devices, CMOS control circuits, and SOS circuits. This all-in-one sapphire platform is designed to achieve high-performance optical links by combining the strengths of various technologies. Additionally, as production scales up, the cost of sapphire is expected to decrease, making the sapphire platform even more attractive.

Waveguide is one of the essential components in PICs, and waveguide loss is a determining factor for overall PIC performance. In this work, silicon nitride straight and bend waveguides on the sapphire platform were investigated via numerical simulation. The refractive index difference of 0.3 between Al_2_O_3_ and Si_3_N_4_ was sufficiently high to build a tightly confined waveguide. For bending waveguides, propagation losses can be suppressed to below 0.01 dB with a bending radius of tens of microns. Since straight and bend waveguides are basic building blocks for passive devices, the results reported in this work provide a baseline for the overall performance of passive components on a sapphire substrate.

## 2. Simulation Method

**Figure 2:**
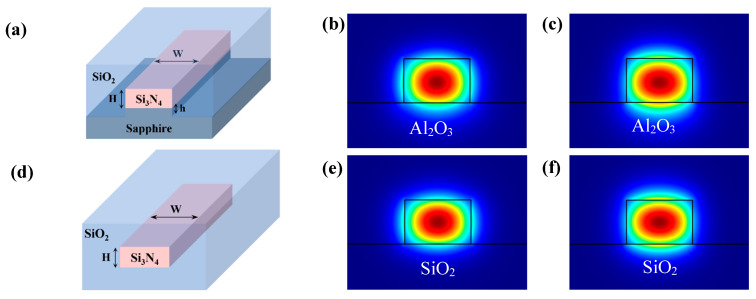
(**a**,**d**) Cross-section of the Silicon nitride waveguide on sapphire and silica. (**b**,**c**) Mode intensity profiles for TE and TM polarization of the Si_3_N_4_/Al_2_O_3_ waveguide. (**e**,**f**) Mode intensity profiles for TE and TM polarization of the Si_3_N_4_/SiO_2_ waveguide.

Commercial software Comsol Multiphysics 6.1 was used to carry out the simulation of the silicon nitride waveguide. Figure 2a illustrates the waveguide structure employed in the simulation. The waveguide consisted of a Si_3_N_4_ ridge on a pedestal of Al_2_O_3_, with a height of h = 0 or 1/2 H on the Al_2_O_3_ substrate. For all the structures, an aspect ratio of W = 1.5 H was selected to model the waveguide, and SiO_2_ was chosen as the over cladding for the simulation. The mode intensity profiles of Si_3_N_4_/Al_2_O_3_ (SiO_2_) waveguides simulated with dimensions of 800 nm ×1200 nm and a wavelength of 1500 nm are shown in Figure 2. Upon comparing the mode intensity profiles under different substrates, we observed that the optical field of the waveguide on the sapphire substrate extended further into the lower cladding layer. The mode area of waveguides on the sapphire substrate was about 7% larger than that of waveguides on the SiO_2_ cladding layer. In addition, the power confinement factor (PCF) of Si_3_N_4_/Al_2_O_3_ was about 3% and 4.5% lower than that of Si_3_N_4_/SiO_2_ for TE and TM modes. This is due to the smaller refractive index difference. Although the confinement ability of the waveguides on the sapphire substrate was slightly reduced, it still provides comparable confinement.

### 2.1. Straight Waveguide

**Figure 3:**
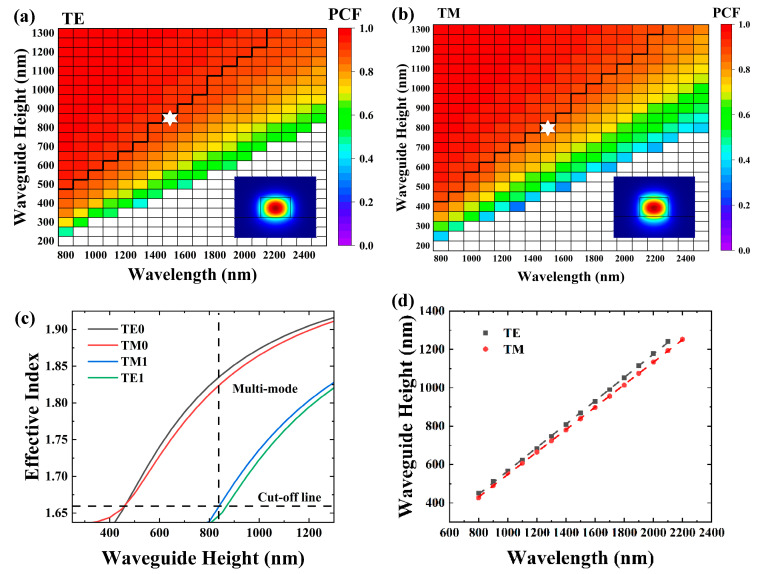
Simulated guided-mode maps for the Si_3_N_4_ waveguide with the general structure shown in Figure 2. W = 1.5 H and h = 0 for the following cases: (**a**) TE polarization and (**b**) TM polarization. The colormaps in the plots represent the power confinement factor (PCF) in the Si_3_N_4_ waveguide. Single-mode, multi-mode, and cut-off regions are marked in each figure. The insets in each plot show the waveguide mode intensity profile at the white star marks. (**c**) Waveguide height-dependent effective index. (**d**) Single-mode limit as a function of wavelength. The dashed lines represent the linear fit.

In order to further investigate the properties of the Si_3_N_4_ waveguides on the sapphire substrate, a detailed analysis of the waveguide modes across a broad optical spectrum, ranging from 800 nm to 2500 nm, was conducted by systematically varying the waveguide width and height. The calculated TE and TM modes for the Si_3_N_4_ waveguide (with W = 1.5 H and h = 0) on a sapphire substrate are shown in Figure 3a and Figure 3b, respectively. The colormaps illustrate the PCF within the Si_3_N_4_ waveguide. Within each plot, the multi-mode, single-mode, and cut-off regions are marked. The black lines represent the boundaries between single-mode and multi-mode operation. The insets located at the corners depict the mode intensity profiles of the waveguides at the position marked by the white star. The PCF of both TE and TM modes exhibits a rapid increase with the expansion of the waveguide size. As the single-mode limit was approached, the PCF of the TE mode reached approximately 89%, whereas the PCF for the TM mode was around 86%. These values indicate that single-mode propagation with strong mode confinement can be achieved. Furthermore, we observed that the PCF of the TE mode was larger than that of the TM mode at the same size. This discrepancy indicates that the confinement of the TE mode was stronger than that of the TM mode. We attribute this phenomenon to the variations in refractive index differences and size across different directions.

The mode characteristics of waveguides can be described by the relationship between the effective index of each mode and the waveguide size. Figure 3c illustrates the effective index as a function of the waveguide size at a wavelength of 1500 nm. The vertical dashed line marks the boundary of the single-mode and multi-mode regions. As the waveguide size increases, the effective index of each mode increases, and the higher order modes become evident.

As shown in Figure 3d, the simulations for the single-mode limit reveal a linear trend. The dashed lines represent the linear fits, with slopes of 0.609 and 0.586 for the TE and TM modes, respectively.

**Figure 4:**
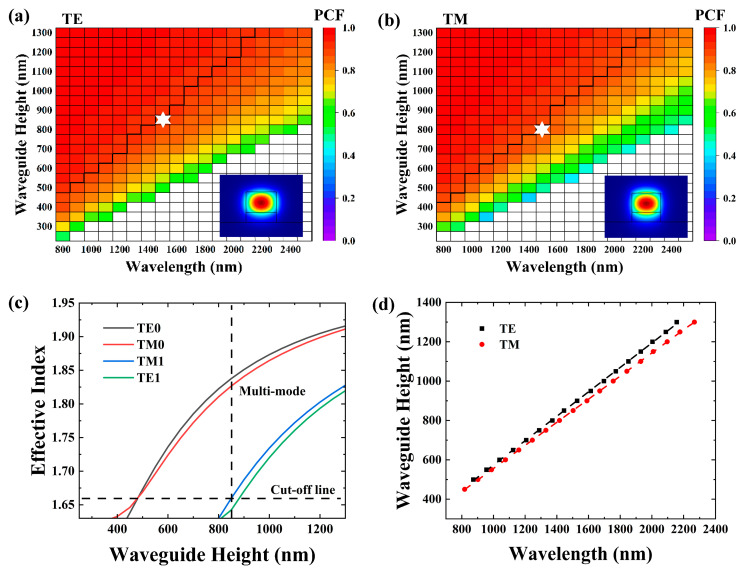
Simulated guided-mode map for the Si_3_N_4_ waveguide with the general structure shown in Figure 2. W = 1.5 H and h = ½ H for the following cases: (**a**,**b**) TE and TM polarization. (**c**) Waveguide height-dependent effective index. (**d**) Single-mode condition as a function of wavelength.

We conducted further simulations for the structure with a pedestal height of h = 1/2 H. Figure 4 illustrates the PCF, effective index, and the single-mode condition for this configuration.

As shown in Figure 4a,b, the PCF near the cut-off region exhibited a slightly higher value compared to that in Figure 3a,b. However, in the high confinement regions, the PCF remained consistent with the values observed in Figure 3a,b. It is worth mentioning that the effective index near the cut-off line in Figure 4c is slightly larger than that in Figure 3c, indicating that the waveguide has better confinement, as observed in Figure 4a,b. The single-mode limit curves in Figure 4d exhibited a similar slope to those observed in Figure 3d, with values of 0.620 and 0.587 for the TE and TM modes, respectively.

**Figure 5:**
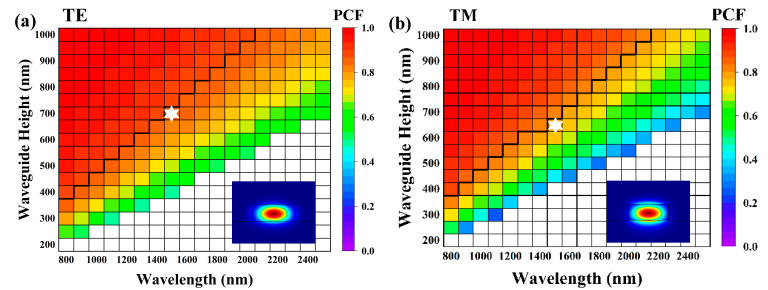
Simulated guided-mode maps for the structure with W = 2 H and h = 0. (**a**) TE and (**b**) TM polarizations.

In order to investigate the effect of the aspect ratio on the waveguide, simulations were performed for the structure with W = 2 H and h = 0. The guided-mode maps are shown in Figure 5. It is intuitive to see that the single-mode limit occurs at a smaller waveguide height, which was expected since the structure has a wider width for the same height. Additionally, compared to the structure with W = 1.5 H, a smaller PCF of about 85% and 80% for TE and TM polarizations, respectively, near the single-mode limit can be observed. Obviously, the thin structure limits its confinement ability, as has been reported in other works [33].

**Figure 6:**
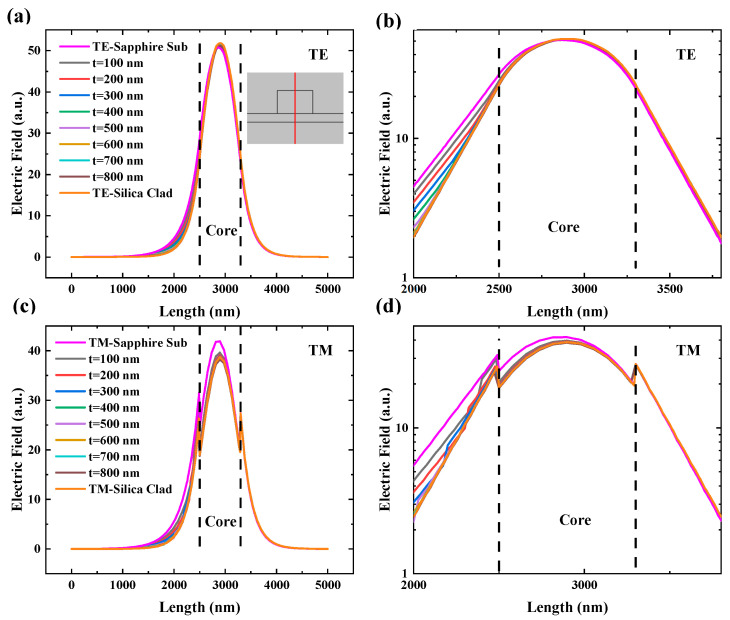
Electric field profiles at the waveguide cross-section for (**a**) the TE mode and (**c**) the TM mode. The insert shows the location of the cross-section (red line), and (**b**,**d**) are zoomed-in views near the core.

To investigate further into the performance of Si_3_N_4_ waveguides on a sapphire substrate, additional simulations were conducted. Additional SiO_2_ bottom cladding layers with varying thicknesses were introduced between the Si_3_N_4_ core and the sapphire substrate. Mode intensity profiles and PCF were then analyzed across different thicknesses of the SiO_2_ bottom cladding layer. To provide a more intuitive observation, the cross-sectional field distribution was used to show the electric field distribution. Figure 6 shows the electric field profile at the waveguide cross-section, with a waveguide size of 800 nm × 1200 nm and a simulated wavelength of 1500 nm. Figure 6a shows the electric field profile for the TE mode. To provide a clearer comparison, Figure 6b shows a zoomed-in view of the core region on a logarithmic scale. As shown in Figure 6a,b, a noticeable mode peak shift was observed in the waveguide without an SiO_2_ bottom cladding layer. Meanwhile, in the lower cladding layer, there was noticeable broadening of the electric field distribution towards the sapphire, whereas this phenomenon was less pronounced within the core. We attribute this to the lower refractive index of sapphire. However, in all waveguides with a SiO_2_ bottom cladding layer, regardless of the SiO_2_ layer thickness, the peaks aligned at the core center, with a symmetric mode field distribution within the core area. Additionally, the broadening in the lower cladding layer gradually decreased with an increase in the thickness of the SiO_2_ layer. Figure 6c shows the electric field profile of the TM mode, and Figure 6d provides a zoomed-in view of the core region. Similar shift and broadening can be observed in Figure 6a,b, which were also caused by the lower refractive index of sapphire. Additionally, the introduction of SiO_2_ effectively mitigated this shift and broadening. This phenomenon indicates that the impact of the lower refractive index of sapphire on the mode field within the core area can be effectively mitigated by the presence of the SiO_2_ layer, even with a small thickness. Additionally, two extra peaks emerged on both sides of the main peak, attributed to the abrupt change in refractive index at the interface between the core and cladding layer. These extra peaks were not observed in the TE mode due to the different polarization directions of the two modes. However, if the cross-sectional direction was rotated by 90°, the peaks appeared in the TE mode instead of the TM mode.

**Figure 7:**
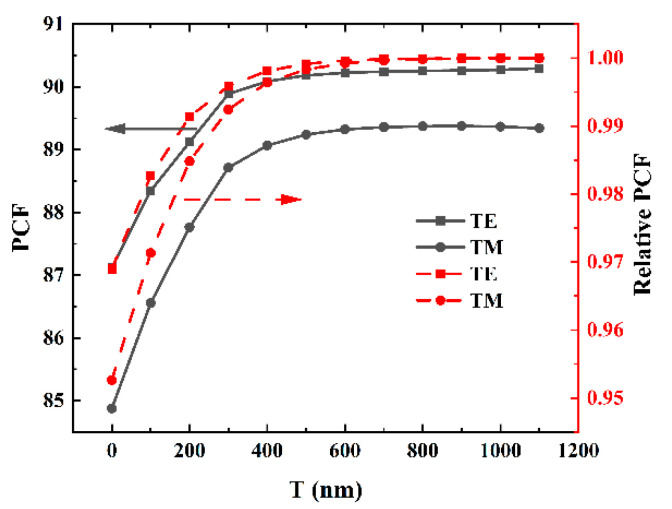
The PCF (black curves) and relative PCF (red curves) of waveguides with varying thicknesses of the SiO_2_ bottom cladding layer. The relative PCF refers to the ratio of the PCF in the calculated structures to that of the Si_3_N_4_/SiO_2_ waveguide.

Figure 7 illustrates the PCF (black curves) of waveguides with different thicknesses of SiO_2_ bottom cladding layers. To enable clear comparison with Si_3_N_4_/SiO_2_ waveguides, the relative PCF (red curves) was also added to the figure, showing the ratio of the PCF of the calculated structure to that of the Si_3_N_4_/SiO_2_ waveguide. As depicted in Figure 7, the PCF increased significantly with increasing SiO_2_ thickness until reaching 400 nm, after which it exhibited a saturation trend beyond 600 nm. The same trend was observed for the relative PCF, indicating that once the thickness of the SiO_2_ layer reached 600 nm, the performance of the waveguide was considered to be consistent with that of the Si_3_N_4_/SiO_2_ structure. In other words, the impact of the sapphire substrate on waveguide performance became negligible. The disparity between the TE and TM modes stems from the differences in the refractive index changes along different directions, as well as the variations in size.

Overall, concerning light confinement, the Si_3_N_4_ waveguide on a sapphire substrate demonstrates performance similar to that of a Si_3_N_4_/SiO_2_ waveguide, even in the absence of the SiO_2_ bottom cladding layer.

### 2.2. Bending Loss

The propagation loss of a waveguide is influenced by several factors, including material absorption, scattering loss, substrate leakage, bend loss, and more [33,34,35]. Due to the low optical absorption of Si_3_N_4_ and Al_2_O_3_, along with continuous advancements in material technology, material absorption loss is minimal and often considered negligible. Since the substrate in this structure also functions as the bottom cladding layer, substrate leakage is not a concern. Scattering loss primarily arises from the roughness of the sidewalls and the top/bottom interfaces [33,34,36,37]. Indeed, although the top and bottom walls can be polished, sidewall roughness emerges as the primary factor contributing to scattering loss. Fortunately, the refractive index of Si_3_N_4_ is close to that of SiO_2_, which makes Si_3_N_4_ more tolerant of process-induced defects and waveguide sidewall roughness. Furthermore, increasingly advanced fabrication processes lead to better sidewalls, facilitating the achievement of lower propagation losses. The outstanding performance of the Si_3_N_4_/SiO_2_ waveguide supports this perspective, not to mention that the performance of the waveguide can be made consistent with the conventional structure by adding the SiO_2_ cladding layer. Bending waveguides can change the direction of light propagation and are an indispensable part of PICs. Evaluating bending loss is essential for optimizing waveguide performance. In addition to the loss factors present in straight waveguides, bending waveguides have two additional major sources of loss: radiation loss caused by mode leakage into the cladding and mismatch loss caused by the mismatch of mode field distributions. In a highly confined waveguide, mode leakage into the cladding in a curved waveguide is typically very small. Instead, the mismatch of mode field distributions between straight and bending waveguides is the primary source of losses in bending waveguides, especially when the bend radius is small [38]. In this work, we primarily focused on calculating mismatch loss in bending waveguides, whereas other losses, such as those caused by material quality and process-induced sidewall roughness, were not included.

**Figure 8:**
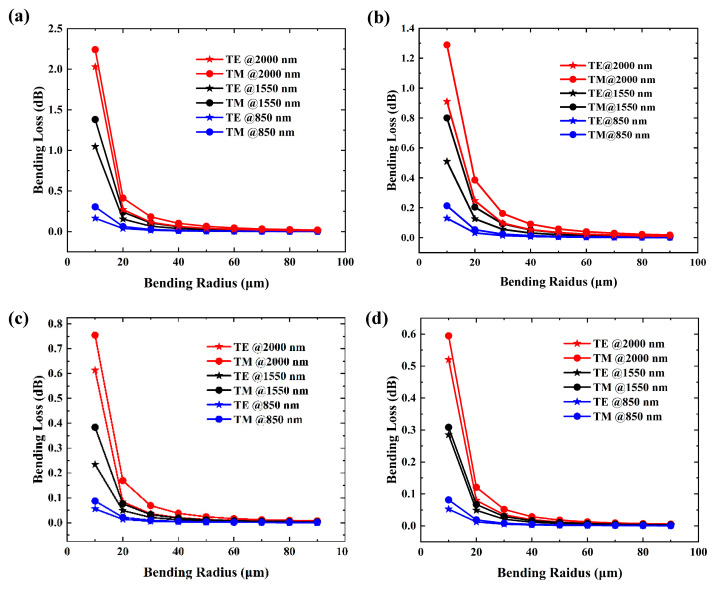
Bending loss near the single-mode limit for (**a**) the Si_3_N_4_ waveguide on sapphire without a pedestal, (**b**) with a pedestal of h = 1/2 H, (**c**) the Si_3_N_4_ waveguide on sapphire with an additional SiO_2_ bottom cladding layer (600 nm), and (**d**) the Si_3_N_4_/SiO_2_ structure at different wavelengths.

Figure 8 illustrates the relationship between bending loss and bending radius. Due to the smaller mode effective area, all structures exhibit lower bending loss at smaller wavelengths. Additionally, losses for TE polarization are smaller than those for TM polarization in both Figure 8a,b, attributed to the differences in refractive index for in-plane and out-of-plane orientations. Upon comparing Figure 8a,b, we observed that the loss for the structure with a pedestal of h = 0.5 H was smaller. We attribute this to the higher refractive index of the side SiO_2_ layer. Figure 8c,d demonstrate significantly lower bending loss with the addition of the SiO_2_ bottom cladding layer. The stronger limiting effect contributes to the reduction of bending losses. Comparing Figure 8c,d, bending losses are generally consistent between these two structures, indicating that the SiO_2_ bottom cladding layer effectively eliminates the influence of the larger refractive index of the substrate, consistent with the results observed in Figure 6 and Figure 7. Meanwhile, the results shown in Figure 8d align with previous reports, validating the findings of this work [39,40,41].

Compared with the basic waveguide structure directly on the sapphire substrate, introducing a pedestal or a SiO_2_ bottom cladding layer effectively reduced bending losses. Even without these modifications, the basic structure’s loss was effectively suppressed as the bending radius reached tens of microns. As depicted in Figure 8a, the bending losses decreased to ~0.01 dB after a bending radius of 90 μm for a wavelength of 2000 nm, 70 μm for 1550 nm, and 40 μm for 850 nm.

## 3. Conclusions

Sapphire emerges as a promising platform for photonic integrated circuits (PICs). The simulation analysis of silicon nitride waveguides on sapphire, conducted using Comsol Multiphysics, demonstrates the feasibility of achieving tightly confined and low-loss waveguides. A high power confinement factor of about 90% near the single-mode limit can be achieved, very low bending loss (~0.01 dB) obtainable at a radius of 40~90 μm for different wavelengths. Additionally, leveraging a pedestal structure and a SiO_2_ bottom cladding layer can further reduce bending loss. Introducing a SiO_2_ bottom cladding layer with enough thickness can effectively eliminate the influence of the larger refractive index of the substrate, resulting in performance comparable to Si_3_N_4_/SiO_2_ waveguides. In conclusion, silicon nitride waveguides on sapphire exhibit performance comparable to Si_3_N_4_/SiO_2_ waveguides, offering advantages in low-loss operation and holding potential for further applications in PICs.

## Figures and Tables

**Table 1:** Summary of CTE.

Material	CTE
Sapphire	6.66 × 10^−6^ °C^−1^ parallel 5.00 × 10^−6^ °C^−1^ perpendicular
GaAs	Linear 5.73 × 10^−6^ °C^−1^
AlAs	Linear 5.23 × 10^−6^ °C^−1^
GaSb	Linear 7.75 × 10^−6^ °C^−1^
InP	Linear 4.60 × 10^−6^ °C^−1^
InSb	Linear 4.52 × 10^−6^ °C^−1^
InAs	6.50 × 10^−6^ °C^−1^ at 80 K 5.04 × 10^−6^ °C^−1^ at 300 K
Si	Linear 2.60 × 10^−6^ °C^−1^

## Data Availability

The data supporting the conclusion of this work are available from the corresponding author on reasonable request.
